# Surface modification of ZnIn_2_S_4_ layers to realize energy-transfer-mediated photocatalysis

**DOI:** 10.1093/nsr/nwac026

**Published:** 2022-02-23

**Authors:** Xianshun Sun, Xiao Luo, Sen Jin, Xiaodong Zhang, Hui Wang, Wei Shao, Xiaojun Wu, Yi Xie

**Affiliations:** Hefei National Laboratory for Physical Sciences at the Microscale, University of Science and Technology of China, Hefei 230026, China; Hefei National Laboratory for Physical Sciences at the Microscale, University of Science and Technology of China, Hefei 230026, China; Hefei National Laboratory for Physical Sciences at the Microscale, University of Science and Technology of China, Hefei 230026, China; Hefei National Laboratory for Physical Sciences at the Microscale, University of Science and Technology of China, Hefei 230026, China; Institute of Energy, Hefei Comprehensive National Science Center, Hefei 230031, China; Hefei National Laboratory for Physical Sciences at the Microscale, University of Science and Technology of China, Hefei 230026, China; Institute of Energy, Hefei Comprehensive National Science Center, Hefei 230031, China; Hefei National Laboratory for Physical Sciences at the Microscale, University of Science and Technology of China, Hefei 230026, China; Hefei National Laboratory for Physical Sciences at the Microscale, University of Science and Technology of China, Hefei 230026, China; Hefei National Laboratory for Physical Sciences at the Microscale, University of Science and Technology of China, Hefei 230026, China; Institute of Energy, Hefei Comprehensive National Science Center, Hefei 230031, China

**Keywords:** surface modification, long-lived species, energy transfer, singlet oxygen, photocatalytic sulphoxidation of sulphides

## Abstract

Photocatalytic selective aerobic oxidation reactions are crucial in designing advanced organic intermediates, but suffer from low conversion efficiency. Hence, activating O_2_ to create suitable reactive oxygen species, such as singlet oxygen (^1^O_2_), can significantly increase the yield of desired products. Herein, using ZnIn_2_S_4_ nanosheets as a model system, we build a surface-modified theoretical structure, where a surface-covered non-conductive macromolecular chain, polyvinyl pyrrolidone (PVP), is bound to ZnIn_2_S_4_ and influences the O_2_ adsorption process. PVP on the surface significantly changes the electronic structure and suppresses electron conduction of ZnIn_2_S_4_ nanosheets. Therefore, abundantly photogenerated and long-lived species transfer their energy to physically absorbed O_2_ to efficiently generate ^1^O_2_, which can oxidize sulphides into their corresponding sulphoxides. For sulphoxidation of different sulphides, surface modification brings a 3–9-fold increase in conversion efficiency and high selectivities ≥98%. This study provides a feasible way of boosting ^1^O_2_-generation-related photocatalytic reactions.

## Teaser

Surface modification strategy of non-conductive PVP chains coating on ZnIn_2_S_4_ nanosheets can inhibit electron transfer and facilitate energy transfer process, where the enhanced long-lived states will boost ^1^O_2_ generation and benefit the photocatalytic sulphoxidation of sulphides.

## INTRODUCTION

Singlet oxygen (^1^O_2_) comes from the triplet state (^3^Σ) of common molecular oxygen (O_2_) and displays its two lowest excited singlet states, ^1^Σ and ^1^Δ, which need excitation energies of 1.63 and 0.98 eV, respectively [1,2]. After its experimental demonstration in 1931, ^1^O_2_ has been developed, with excellent prospects in the fields of photodynamic therapy, water treatment and selective organic synthesis [3–5], where ^1^O_2_ has been shown to be able to react with organic molecules to produce value-added organic chemicals [1,6–10]. Because of the spin selection rule, direct excitation from the triplet ground-state O_2_ to ^1^O_2_, and chemical reactions between singlet organic molecules and O_2_, are forbidden [2]. Generally, ^1^O_2_ can be obtained via long-lived species or sequential charge transfer processes such as the exciton-related energy transfer process, which is preferred because of its high ^1^O_2_ generation [2,11,12].

However, due to the low concentration of long-lived species in semiconductors, the photosensitizers that have been exploited for ^1^O_2_ generation are primarily limited to molecular dyes, which have the disadvantages of instability and high cost. Although low-dimensional semiconductors with a confined-layer structure were recently reported with enhanced exciton generation [9,13–16], their unique surface structure is beneficial in transporting free charge carriers to absorbed molecular oxygen, thereby weakening the desired energy transfer process. Therefore, using a two-dimensional (2D) confined structure, we have attempted to optimize ^1^O_2_ generation by blocking competitive charge transfer processes [17,18].

**Figure 1.**
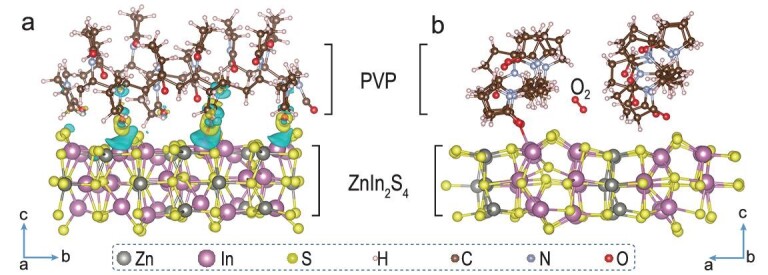
(a) Calculated deformation charge density of PVP adsorbing on ZnIn_2_S_4_. (b) O_2_ adsorbed inside of a PVP-covered ZnIn_2_S_4_ model.

Herein, using a ZnIn_2_S_4_ nanosheet as a model system, we have built a surface modification structure by covering its surface with polyvinyl pyrrolidone (PVP). As a non-conductive polymer [19], the PVP-covered surface can inhibit the electron transfer process from ZnIn_2_S_4_ nanosheets to O_2_. Therefore, theoretical simulations were conducted to study this issue, where a PVP-covered ZnIn_2_S_4_ model and perfect ZnIn_2_S_4_ slab were built for comparison. For a perfect ZnIn_2_S_4_ structure, the adsorption energies of O_2_ at In and Zn sites are −0.883 and −0.138 eV, where 0.143 and 0.057 electrons are transferred to O_2_ via Bader analysis [20–22], respectively (Supplementary Fig. 1). Considering that injected electrons can change the orbital degeneracy of O_2_, we deduce that O_2_ is primarily chemisorbed on In sites rather than Zn sites. After adding PVP onto the ZnIn_2_S_4_ slab, the carbonyl oxygen in PVP stably occupied and saturated In sites with an adsorption energy of −4.188 eV (Fig. 1a). Moreover, due to steric hindrance, O_2_ tends to physically absorb between PVP chains and the ZnIn_2_S_4_ surface by van der Waals interactions, with a negative adsorption energy of −0.220 eV (Fig. 1b and Supplementary Fig. 2). Hence, a PVP-covered surface can effectively prevent chemisorption and electron transfer between O_2_ and ZnIn_2_S_4_.

## RESULTS AND DISCUSSION

**Figure 2.**
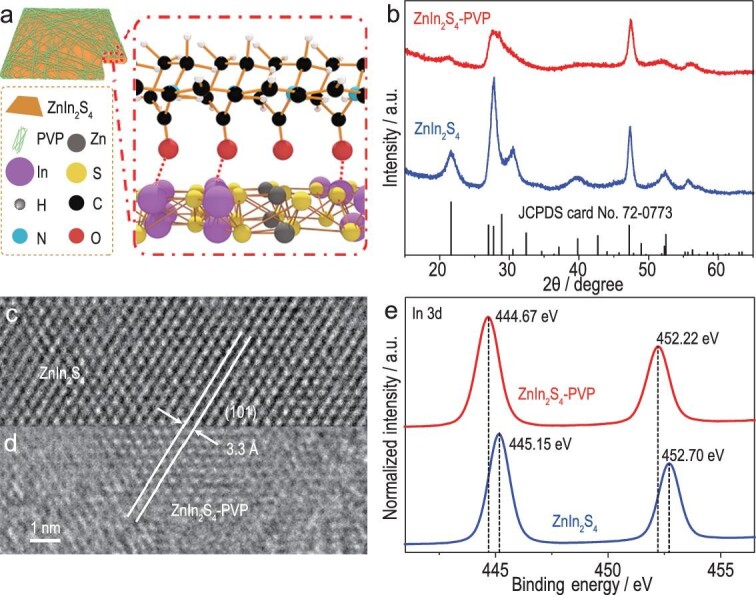
(a) Schematic illustration of PVP-covered ZnIn_2_S_4_. (b) XRD patterns. HRTEM image of (c) pristine ZnIn_2_S_4_ and (d) ZnIn_2_S_4_-PVP, respectively. (e) In 3d XPS spectra.

In this study, PVP-covered ZnIn_2_S_4_ (ZnIn_2_S_4_-PVP) nanosheets (Fig. 2a) and pristine ZnIn_2_S_4_ nanosheets were prepared using a hydrothermal method. Their X-ray diffraction (XRD) characterization demonstrated the high purity of hexagonal ZnIn_2_S_4_ (Joint Committee on Powder Diffraction Standards (JCPDS) card no. 72-0773; Fig. 2b). Moreover, both samples showed sheet-like morphologies, as depicted by scanning electron microscopy and transmission electron microscopy images (Supplementary Fig. 3). In addition, high-resolution transmission electron microscopy (HRTEM) revealed interlayer distances of 3.3 Å, corresponding to the (101) plane of hexagonal ZnIn_2_S_4_ (Fig. 2c and d).

The fine structure of ZnIn_2_S_4_-PVP nanosheets was investigated via Fourier transform infrared (FTIR) spectroscopy, and characteristic peaks similar to PVP were observed (Supplementary Fig. 4) [23]. Meanwhile, energy dispersive spectrometer (EDS) mapping indicated the equally distributed atoms of C, N and O on the surface of ZnIn_2_S_4_ nanosheets (Supplementary Fig. 5), thereby confirming the presence of a surface-covering polymer. Moreover, X-ray photoelectron spectroscopy (XPS) demonstrated the chemical bonding between PVP and ZnIn_2_S_4_. As seen in Fig. 2e and Supplementary Fig. 6, varying degrees of blue shifts were exhibited in Zn 2p (0.54 eV), In 3d (0.48 eV) and S 2p (0.46 eV) XPS spectra of ZnIn_2_S_4_-PVP nanosheets relative to the pristine sample, and were caused by carbonyl oxygen binding to ZnIn_2_S_4_ [24,25]. Additionally, thermogravimetric analysis (TGA) revealed that ZnIn_2_S_4_-PVP nanosheets contain ∼20% PVP (by weight) (Supplementary Fig. 7). These results infer that PVP-covered ZnIn_2_S_4_ nanosheets were successfully prepared.

**Figure 3.**
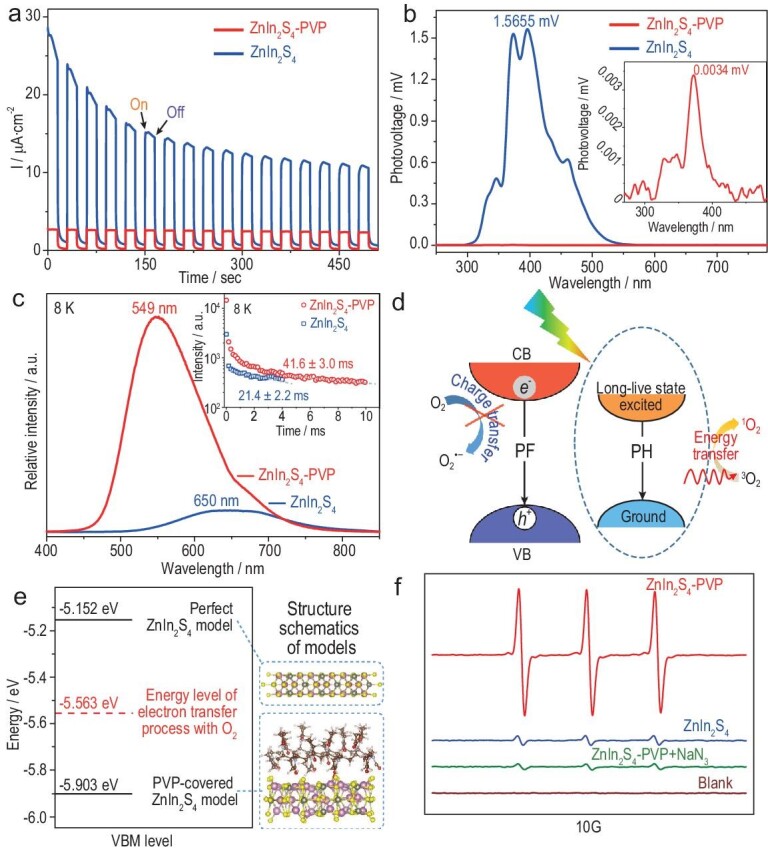
(a) Periodic on/off photocurrent response at 0.6 V. (b) SPV spectra. (c) Low-temperature (8 K) prompt fluorescence (PF) excited at 370 nm (inset: time-resolved PH spectra under 8 K). (d) Schematic illustration of suppression electron transfer and enhancement of ^1^O_2_ generation via energy transfer. (e) Calculated energy positions of the VBM of different models relative to vacuum level. (f) ESR spectra in the presence of TEMP.

PVP coating greatly influences the photocatalytic activity of the sample. For example, in gas molecule absorption–desorption tests, ZnIn_2_S_4_-PVP nanosheets exhibited a Brunauer-Emmett-Teller (BET) surface area of 30.82 m^2^ g^−1^, much smaller than that of pristine ZnIn_2_S_4_ nanosheets (72.85 m^2^ g^−1^; Supplementary Fig. 8a). This result indicates the reduced gas adsorption ability of ZnIn_2_S_4_-PVP nanosheets, where PVP coating blocks the contact between gas molecules and ZnIn_2_S_4_. Furthermore, the photoresponsive behaviour of the samples was studied by the periodic on/off photocurrent (Fig. 3a), in which the transient value of ZnIn_2_S_4_-PVP is stable and less than that of pristine ZnIn_2_S_4_. Surface photovoltage (SPV) analysis (Fig. 3b) of ZnIn_2_S_4_-PVP exhibits an SPV response of 0.0034 mV, which is much less than that of the pristine sample (1.5655 mV). These electrochemical analyses give direct evidence that the PVP coating greatly suppresses the electron transfer process between ZnIn_2_S_4_ and adsorbed O_2_.

To investigate the photogenerated species in ZnIn_2_S_4_-PVP, we performed room-temperature/low-temperature (8 K) prompt fluorescence (PF) and phosphorescence (PH) measurements. As shown in Fig. 3c and Supplementary Fig. 9, ZnIn_2_S_4_-PVP displayed a stronger PF emission peak than pristine ZnIn_2_S_4_ at 570 nm (298 K) and 549 nm (8 K), illustrating the increase in photogenerated species. With a record at 2 ms delay, ZnIn_2_S_4_-PVP and pristine ZnIn_2_S_4_ presented PH emission peaks at ∼600 and ∼700 nm and the corresponding calculated monoexponential lifetimes were ∼41.6 and 21.4 ms, respectively, demonstrating that the photogenerated species are long-lived states. It is known that PH arises from radiative decay of spin-forbidden long-lived states (using a certain delay time to distinguish PF spectra), while PF is induced by spin-allowed radiative decay [26]. Thus, due to the suppressed electron transfer process induced by the PVP coating, massive photoexcited long-lived binding states will be formed in ZnIn_2_S_4_-PVP nanosheets (Fig. 3d).

Furthermore, the valence band maximum (VBM) level (an important part of the electron transfer process) of different systems based on the frontier orbital theory was conducted to thoroughly understand the photogenerated species generation processes affected by PVP coating [27–30]. As illustrated in Fig. 3e, the calculated energy level of the electron transfer process with O_2_ is −5.563 eV. Moreover, the VBM level of the PVP-covered ZnIn_2_S_4_ model is −5.903 eV, indicating that the PVP coating can effectively inhibit the electron transfer process. However, spontaneous electron transfer exists in the perfect ZnIn_2_S_4_ model due to a VBM level of −5.152 eV (much larger than −5.563 eV). The above results indicate the suppression of electron-transfer-dependent O_2_^•−^ generation for ZnIn_2_S_4_ with PVP coating on its surface, suggesting that a competitive energy-transfer-related ^1^O_2_ generation process would be facilitated.

Hence, we deduce that ^1^O_2_ can be obtained by energy transfer from the excited long-lived states of ZnIn_2_S_4_-PVP to ground-state O_2_. To explore the role of PVP coating in ^1^O_2_ generation, electron spin resonance (ESR) trapping measurements were conducted in the presence of 2,2,6,6-tetramethylpiperidine (TEMP), which can trap ^1^O_2_ to show a triplet ESR signal. As expected, ZnIn_2_S_4_-PVP displayed a stronger 1:1:1 triplet signal than pristine ZnIn_2_S_4_, and the added ^1^O_2_ scavenger (NaN_3_) significantly reduced the intensity of the ESR signal (Fig. 3f). Moreover, ZnIn_2_S_4_-PVP had better ^1^O_2_ generation ability under visible-light irradiation than pristine ZnIn_2_S_4_ via the oxidation of 9,10-anthracenediyl-bis(methylene)dimalonic acid (ABDA) (Supplementary Figs 10 and 11) [9,31,32]. Note that the concentration of ^1^O_2_ is not positively related to the covering rate of PVP coating. After adding 400 mg PVP instead of 100 mg at the synthetic section, the sample (90% of PVP in weight) exhibited negligible gas adsorption, as well as limited ^1^O_2_ or superoxide radical (O_2_^•−^) generation. The PVP layers on ZnIn_2_S_4_ nanosheets thoroughly blocked both electron and energy transfer processes between the catalyst and O_2_ (Supplementary Figs 7, 8b, 12 and 13).

Benefiting from the increased concentration of ^1^O_2_, ZnIn_2_S_4_-PVP can be employed to synthesize chemical intermediates of sulphoxides. As shown in Table 1, ZnIn_2_S_4_-PVP had a higher photocatalytic performance than its pristine counterpart for various sulphides under visible-light irradiation. For example, under room-temperature and 1 atm atmospheric pressure, the conversion rate of thioanisole via ZnIn_2_S_4_-PVP is up to 99%, ∼6.6 times greater than that of pristine ZnIn_2_S_4_ (entry 1; Supplementary Fig. 14). With a substituted atmosphere of argon, entry 2 illustrates the vital role of O_2_ in the photocatalytic process. Moreover, the addition of 2-methylfuran, an effective ^1^O_2_ scavenger, significantly suppresses the conversion efficiency of both samples (entry 3), which indicates the reaction based on ^1^O_2_ [33]. Regarding substrates with different sulphides, ZnIn_2_S_4_-PVP showed enhanced conversion efficiency in contrast to pristine ZnIn_2_S_4_ (entries 4−9) with selectivities ≥98%. Furthermore, the ZnIn_2_S_4_-PVP sample appears to be a stable catalyst over 15 catalytic cycles (Supplementary Fig. 15). Hence, for the ^1^O_2_-involved photocatalytic sulphoxidation of sulphides, we have proposed a possible mechanism in Supplementary Fig. 16. In detail, under visible-light irradiation, the physically absorbed ground-state O_2_ on ZnIn_2_S_4_-PVP transforms into ^1^O_2_ by energy transfer, and then reacts with substrates to generate final products. This mechanism also gives a rational explanation of steric hindrance to Supplementary Table 1 if the methyl R_2_ groups are replaced with larger side groups, such as cyclopropyl or phenyl groups, where the conversion rates were shown to be inhibited.

**Table 1.** Sulphoxidation of sulphides^a^.

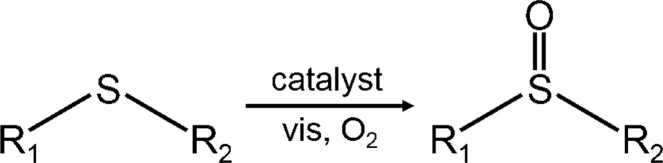							
			ZnIn_2_S_4_-PVP		ZnIn_2_S_4_		
Entry	R_1_	R_2_	Time/h	Con.^b^	Sel.^c^	Con.	Sel.
1	Ph	CH_3_	16	99	99	15	99
2^d^	Ph	CH_3_	16	trace	–	trace	–
3^e^	Ph	CH_3_	16	4.5	99	2.3	99
4	p-MeOPh	CH_3_	5	99	99	36	99
5	o-MeOPh	CH_3_	10	99	99	28	99
6	p-MePh	CH_3_	14	98	99	16	99
7	p-FPh	CH_3_	18	99	98	19	99
8	p-ClPh	CH_3_	24	96	98	11	99
9	p-BrPh	CH_3_	24	91	99	16	99

^a^Reaction conditions: catalyst (20 mg), substrate (0.1 mmol), acetonitrile (4 mL), xenon lamp (300 W) equipped with a 400 nm cut-off filter, 298 K, O_2_ (1 atm). ^b^Determined by nuclear magnetic resonance (NMR) analyses using dichloromethane as the internal standard, mol %. ^c^Selectivity = yield/conversion, mol %. ^d^Ar, 1 atm. ^e^Additional 2-methylfuran (400 μL).

## CONCLUSIONS

In summary, we have proposed an energy transfer process mediated by surface modification to boost ^1^O_2_ generation in ZnIn_2_S_4_ nanosheets via a PVP coating on their surface. Systematic experiments and theoretical simulations indicate that the PVP coating efficiently inhibits the electron transfer process from ZnIn_2_S_4_ to physically absorbed O_2_ and boosts the long-lived binding states, resulting in the generation of ^1^O_2_ via energy transfer. Benefiting from the enhanced ^1^O_2_ generation, ZnIn_2_S_4_-PVP’s photocatalytic selective oxidation performance under visible-light irradiation was excellent. This work offers a universal approach for designing advanced materials with efficient energy-transfer-dependent ^1^O_2_ generation and photocatalytic aerobic selective oxidation reactions.

## METHODS

### Calculations

DFT calculations were performed using the Vienna Ab-initio Simulation Package (VASP) with the projector augmented wave method for the core region and a plane-wave kinetic energy cut-off of 480 eV [34–36]. The generalized gradient approximation method, with Perdew–Burke–Ernzerh (PBE) functional for the exchange-correlation term, was used. The convergence of energy and force were set to less than 1 × 10^−5^ eV and 0.05 eV/Å, respectively. The three atomic layers along the [110] projection were used to simulate the surface of nanosheets, and a 2.0 nm vacuum layer was added to avoid interaction between adjacent layers.

To match the periodic lattice of PVP and ZnIn_2_S_4_, supercells of 1 × 3 ZnIn_2_S_4_ [110] unit cells were used to load supercells of 2 × 6 PVP unit cells. A set of 2 × 2 × 1 k-points were sampled using a gamma-centred Monkhorst–Pack scheme to describe the Brillouin zone. Charge distributions were analysed via Bader's model [20–22]. The differential charge density was plotted with an isosurface value of 0.002 e/Bohr^−3^. Adsorption energy was calculated as follows:

$$\begin{equation*}
{E_{ads}} = {E_{aft_ads}} - {E_{base_ads}} - {E_{{speci_ads}}}.
\end{equation*}$$

***E_aft_ads_*** represents the energy of the most stable adsorption model in optimization, ***E_base_ads_*** represents the energy of the most stable structure of different bases in optimization and ***E_speci_ads_*** represents the energy of adsorbed species, such as PVP, NVP and oxygen.

## Supplementary Material

nwac026_Supplemental_File
