# Supplementary material for: Surface modification of ZnIn2S4 layers to realize energy-transfer-mediated photocatalysis
Source: Natl Sci Rev. 2022 Feb 23;9(10):nwac026. doi: 10.1093/nsr/nwac026 (PMC9671662; doi:10.1093/nsr/nwac026)
Supplement: nwac026_Supplemental_File [file nwac026_supplemental_file.doc]

Supporting Information

**Surface modification of ZnIn_2_S_4_ layers to realise energy transfer-mediated photocatalysis**

Xianshun Sun^1,†^, Xiao Luo^1,†^, Sen Jin^1^, Xiaodong Zhang^1,2^,*, Hui Wang^1,2^, Wei Shao^1^, Xiaojun Wu^1^,* and Yi Xie^1,2^,*

1. Hefei National Laboratory for Physical Sciences at the Microscale, University of Science and Technology of China, Hefei 230026, China

2. Institute of Energy, Hefei Comprehensive National Science Center, Hefei 230031, China

***Corresponding authors**

E-mail: zhxid@ustc.edu.cn; xjwu@ustc.edu.cn; yxie@ustc.edu.cn

**Author contributions:** †Equally contributed to this work.

**Experimental Sections**

**Synthesis**

**PVP-covered ZnIn_2_S_4_ (ZnIn_2_S_4_-PVP) Nanosheets:** In the synthetic process, 0.4 mmol of ZnCl_2_, 0.8 mmol of InCl_3_•4H_2_O, 3.2 mmol of thioacetamide, and 100 mg of polyvinyl pyrrolidone (PVP, K23-27, average molecular weight: ~24000) were dissolved in a mixed solution of 15 mL of ethanol and 15 mL of water. With vigorous stirring for 30 min, the solution was transferred into a 40 mL Teflon cup, then sealed in the autoclave and kept under 180 °C for 24 h. After naturally cooled down, the suspension was collected and washed with distilled water and ethanol three times, and then dried at 60 °C in air overnight for further analysis.

**Pristine ZnIn_2_S_4_ (ZnIn_2_S_4_) Nanosheets:** The synthetic process is same to the synthesis of ZnIn_2_S_4_-PVP nanosheets without adding of polyvinyl pyrrolidone.

**Over PVP-covered ZnIn_2_S_4_ (ZnIn_2_S_4_-over-PVP) Nanosheets:** The synthetic process is same to the synthesis of ZnIn_2_S_4_-PVP nanosheets except increasing the amount of PVP to 400 mg.

**Electron Spin Resonance Trapping Measurements:** 100 *μ*L of the aqueous suspension of the sample (4 g L^−1^) was mixed with 500 *μ*L of trapping agent solution (50 mM). After being irradiated for 1 min with visible light, the mixed solution was characterized by using a JEOL JES-FA200 electron spin resonance spectrometer (298 K, 9.062 GHz).

**Singlet Oxygen Detection by Chemical Oxidation of 9,10-anthracenediyl-bis(methylene)dimalonic Acid (ABDA):** 5.1 mg of ABDA and 40 mg of catalyst were spread to 50 mL of phosphate buffer (pH = 6.86) in a quartz beaker by sonicating for a few seconds. After bubbling with oxygen/nitrogen for 60 s, sealing the beaker and irradiating under a xenon lamp equipped with a 400 nm cut-off filter with vigorous stirring. Sampling the aqueous suspension every 2 min and collecting the solution by filter. The oxidation of ABDA were evaluated by UV−Vis measurements (the absorbance from ~320 nm to ~450 nm) at different time intervals.

**Visible** **Light-Driven Photocatalytic Measurements for Sulphoxidation of Sulphides:** Firstly, 20 mg of catalyst was spread to 4 mL of acetonitrile in a quartz tube (10 mL) by sonicating for a few seconds. Secondly, adding substrate into the mixed solution and bubbling with oxygen for 30 s. Then sealing and irradiating the quartz tube by a 300 W xenon lamp (PLS-SXE300/300UV, Trusttech Co., Ltd., Beijing) equipped with a 400 nm cut-off filter with vigorous stirring for hours at 298 K. Finally, the obtained solution was collected by centrifugation and determined by ^1^H NMR spectroscopy using dichloromethane as the internal standard substance.

**Characterizations**

Powder X-ray diffraction patterns (PXRD) were carried out on a Japan Rigaku MiniFlex 600 equipped with graphite-monochromated Cu K*α* radiation (*λ* = 1.54178 Å). Transmission electron microscopy (TEM) images were recorded on a Hitachi-H7650 transmission electron microscope. Scanning electron microscopy (SEM) images were taken on a FEI Sirion-200 field emission scanning electron microscope operated at 5 kV. High-resolution transmission electron microscopy (HRTEM) images and corresponding energy dispersive spectroscopy (EDS) mapping analyses were recorded on a JEM-2100F field-emission electron microscope at an acceleration voltage of 200 kV. X-ray photoelectron spectroscopy (XPS) valence spectra were acquired on an ESCALAB MKII spectrometer with a Mg K*α* excitation source (*hν* = 1253.6 eV). The binding energies derived from the XPS analysis were corrected against the specimen charging by referencing C 1s to 284.8 eV. UV−vis spectra were acquired on a PerkinElmer Lambda 950 UV−vis−NIR spectrophotometer. Photoluminescent (PL) spectra were acquired on a Hitachi F-4600 spectrometer. Time-resolved fluorescence spectra were measured on HORIBA HuoroMax-4P. Fourier transform infrared (FT-IR) spectra were obtained on a Magna-IR750 FT-IR spectrometer in a KBr pellet, scanning from 4000 to 400 cm^−1^ at room temperature. Room-temperature electron spin resonance (ESR) spectra were collected using a JEOL JES-FA200 electron spin resonance spectrometer (298 K, 9.062 GHz). Gas sorption measurement was conducted by using an automatic volumetric adsorption equipment (Micrometritics ASAP 2020). Thermogravimetric Analysis (TGA) were carried out on a TGA Q5000 device with a heating rate of 10 °C min^−1^ under the atmosphere of N_2_. ^1^H NMR experiments were performed with a 400 MHz Bruker AVANCE AV III NMR spectrometer. The SPV spectra were obtained by measurement system including a lockin amplifier (SR830, Stanford research systems, Inc.), monochromatic-light, a light chopper (SR540, Stanford research systems, Inc.), and a simple chamber. Monochromatic light is generated from a 500 W xenon lamp (CHF-XM-500 W, global xenon lamp power) by a monochromator (Omni-3007, No.16047, Zolix). The electrochemical measurements were performed on an electrochemical workstation (CHI760E, Shanghai Chenhua Limited, China).


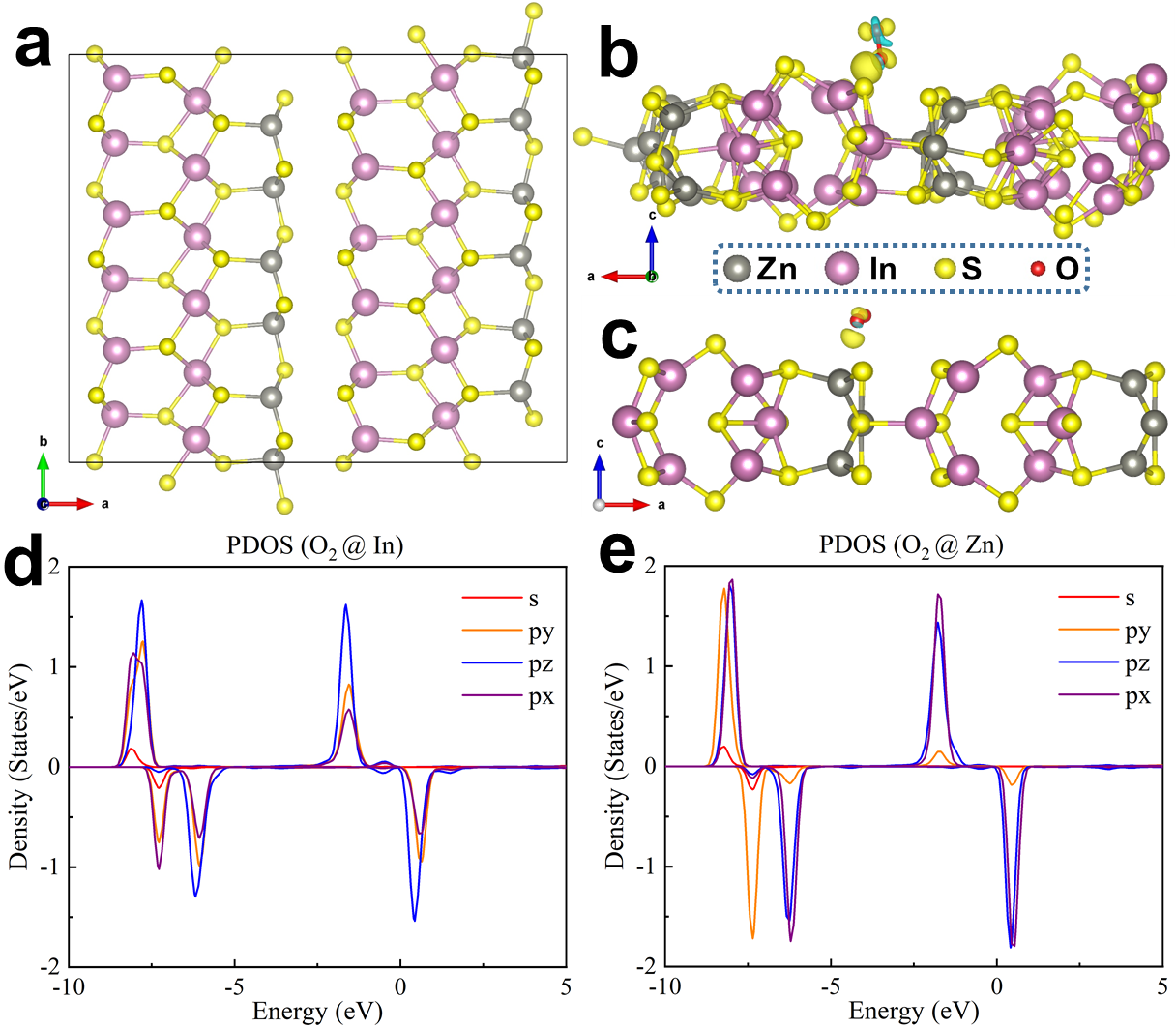


**Supplementary Figure 1.** (a) Structure of perfect ZnIn_2_S_4_ model from the [110] orientation view. Calculated deformation charge density of O_2_ adsorbing on (b) In and (c) Zn of ZnIn_2_S_4_. Calculated DOS of O_2_ adsorbing on (d) In and (e) Zn of ZnIn_2_S_4_.


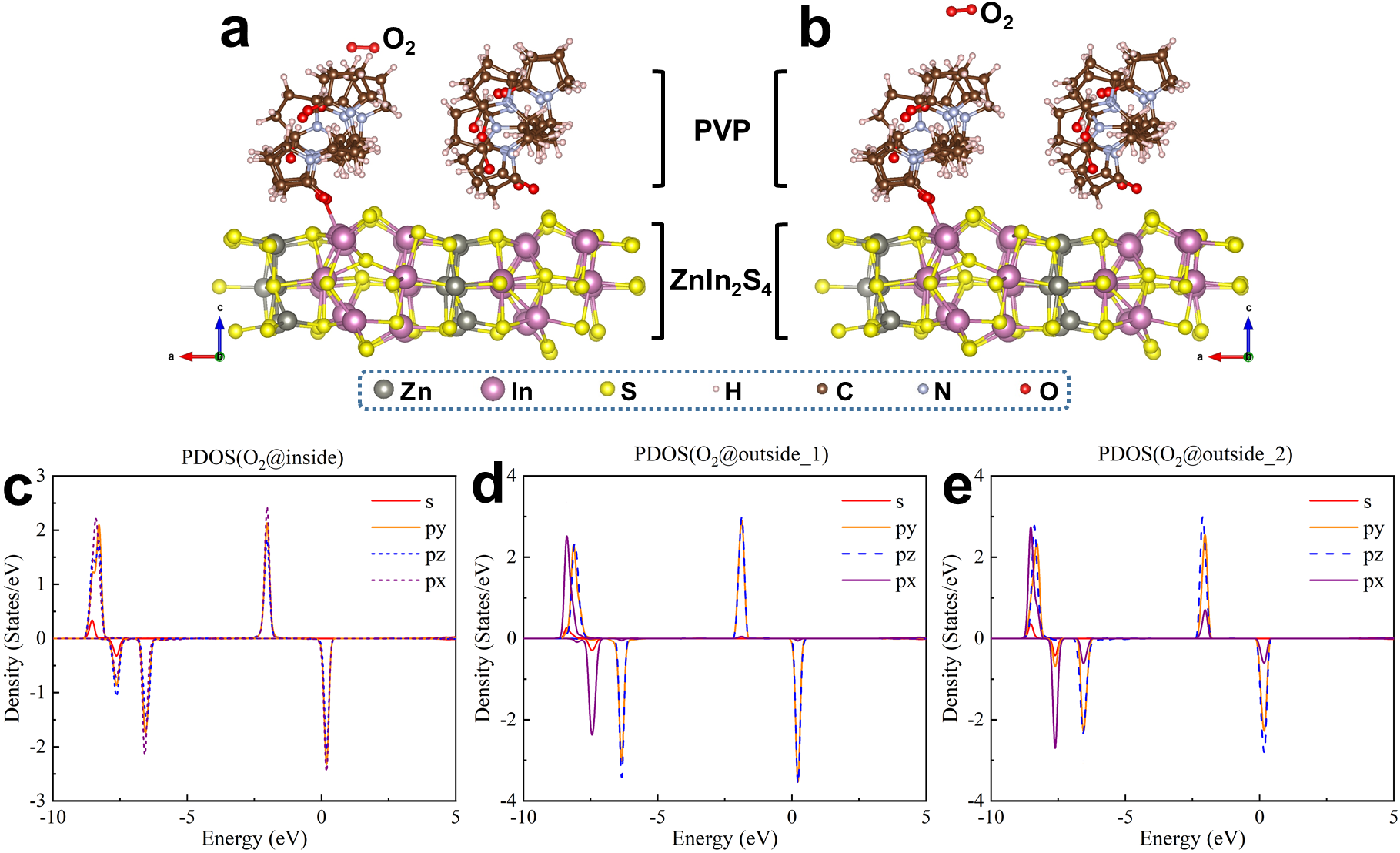


**Supplementary Figure 2.** Calculated deformation charge density of O_2_ adsorbing outside PVP-covered ZnIn_2_S_4_ model: (a) O_2_@outside_1 and (b) O_2_@outside_2. Calculated DOS of O_2_ adsorbing: (c) O_2_@inside, (d) O_2_@outside_1 and (e) O_2_@outside_2. For model of O_2_@outside_1 and O_2_@outside_2, the O_2_ adsorption energy are -0.137 and -0.064 eV, respectively.


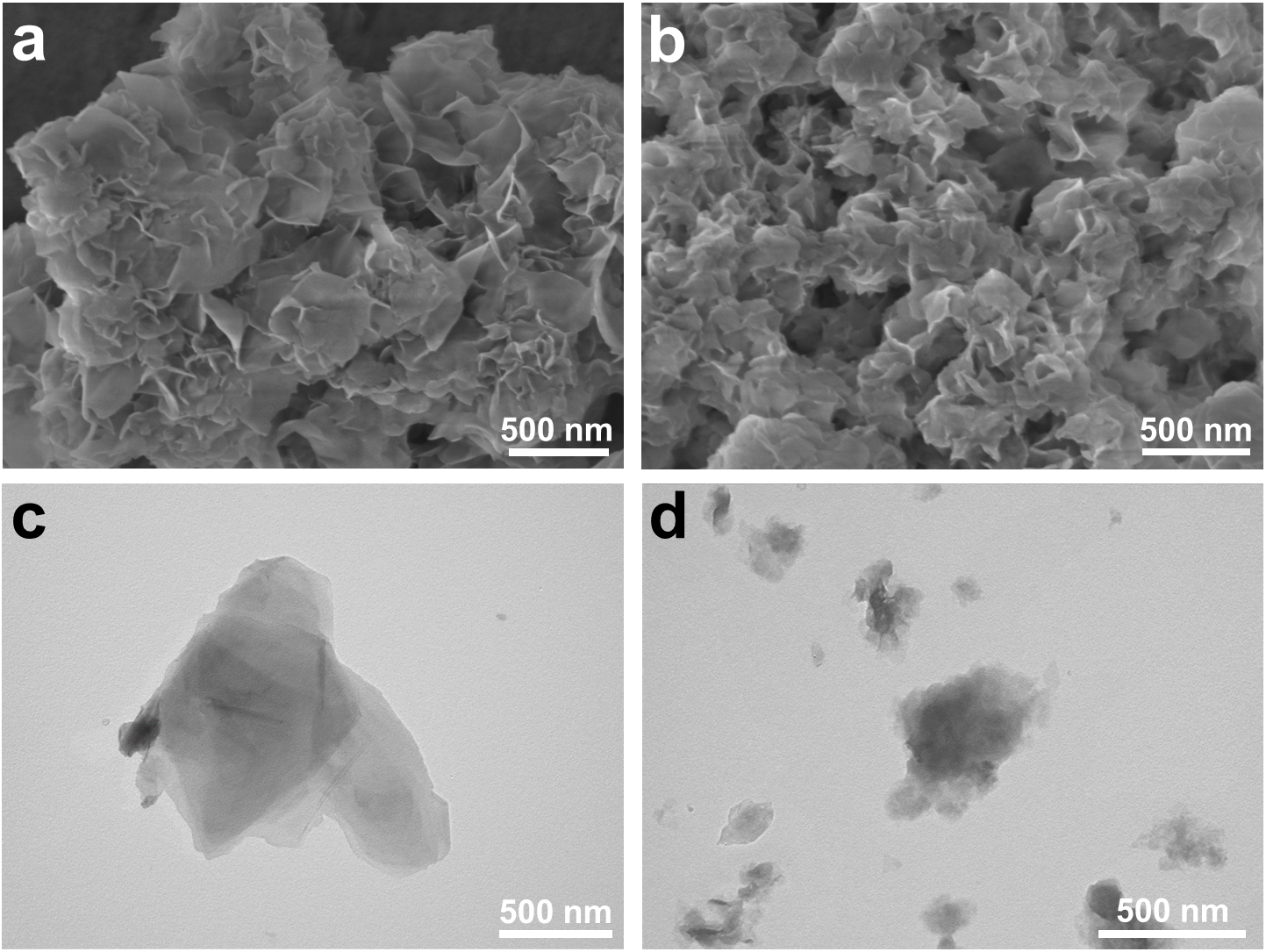


**Supplementary Figure 3.** SEM images of (a) ZnIn_2_S_4_ and (b) ZnIn_2_S_4_-PVP. TEM images of (c) ZnIn_2_S_4_ and (d) ZnIn_2_S_4_-PVP.


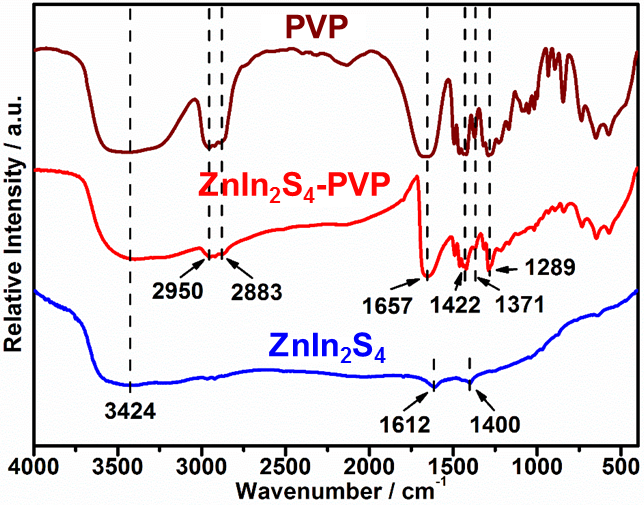


**Supplementary Figure 4.** FT-IR spectra of ZnIn_2_S_4_-PVP, ZnIn_2_S_4_, and PVP. As for the ZnIn_2_S_4_, the peaks at around 3424, 1612, and 1400 cm^−1^ correspond to the surface adsorbed water and hydroxyl groups, respectively. In comparison, both pure PVP and ZnIn_2_S_4_-PVP show similar new absorption bands at 2950, 2883, 1657, 1422, 1371, and 1289 cm^−1^, which can be assigned to the asymmetric CH_2_ stretching vibration (2950 cm^−1^), symmetric CH_2_ stretching vibration (2883 cm^−1^), C=O stretching vibration (1657 cm^−1^), CH deformation modes from the CH_2_ group (1422 and 1371 cm^−1^), and C–N bending vibration (1289 cm^−1^), respectively [1], proving the existence of PVP in ZnIn_2_S_4_-PVP.


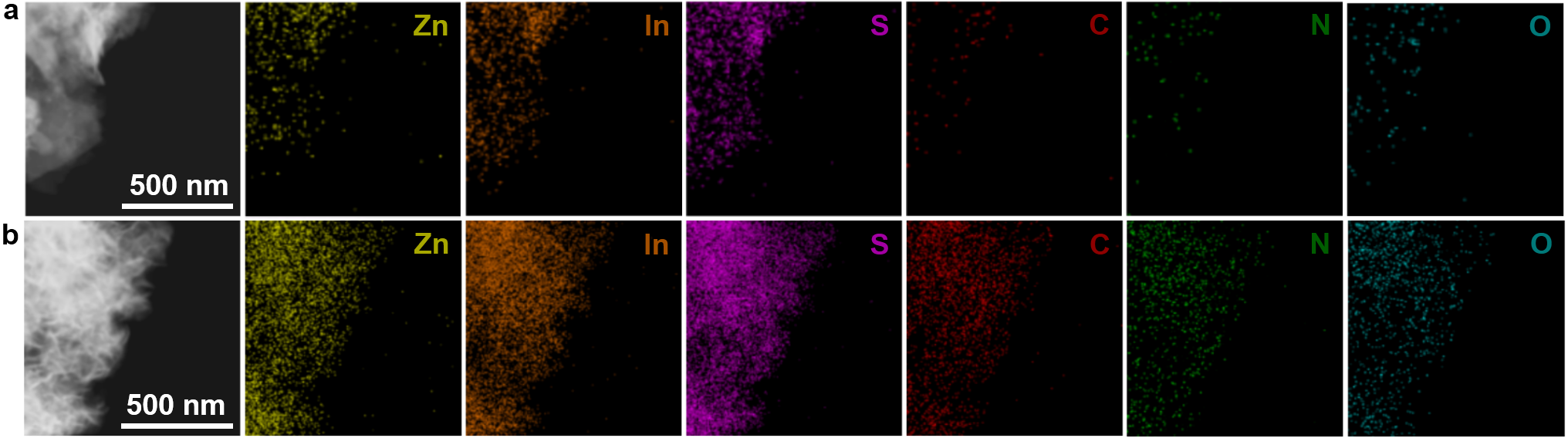


**Supplementary Figure 5.** HAADF-STEM images and corresponding EDS mapping images of (a) ZnIn_2_S_4_ and (b) ZnIn_2_S_4_-PVP.


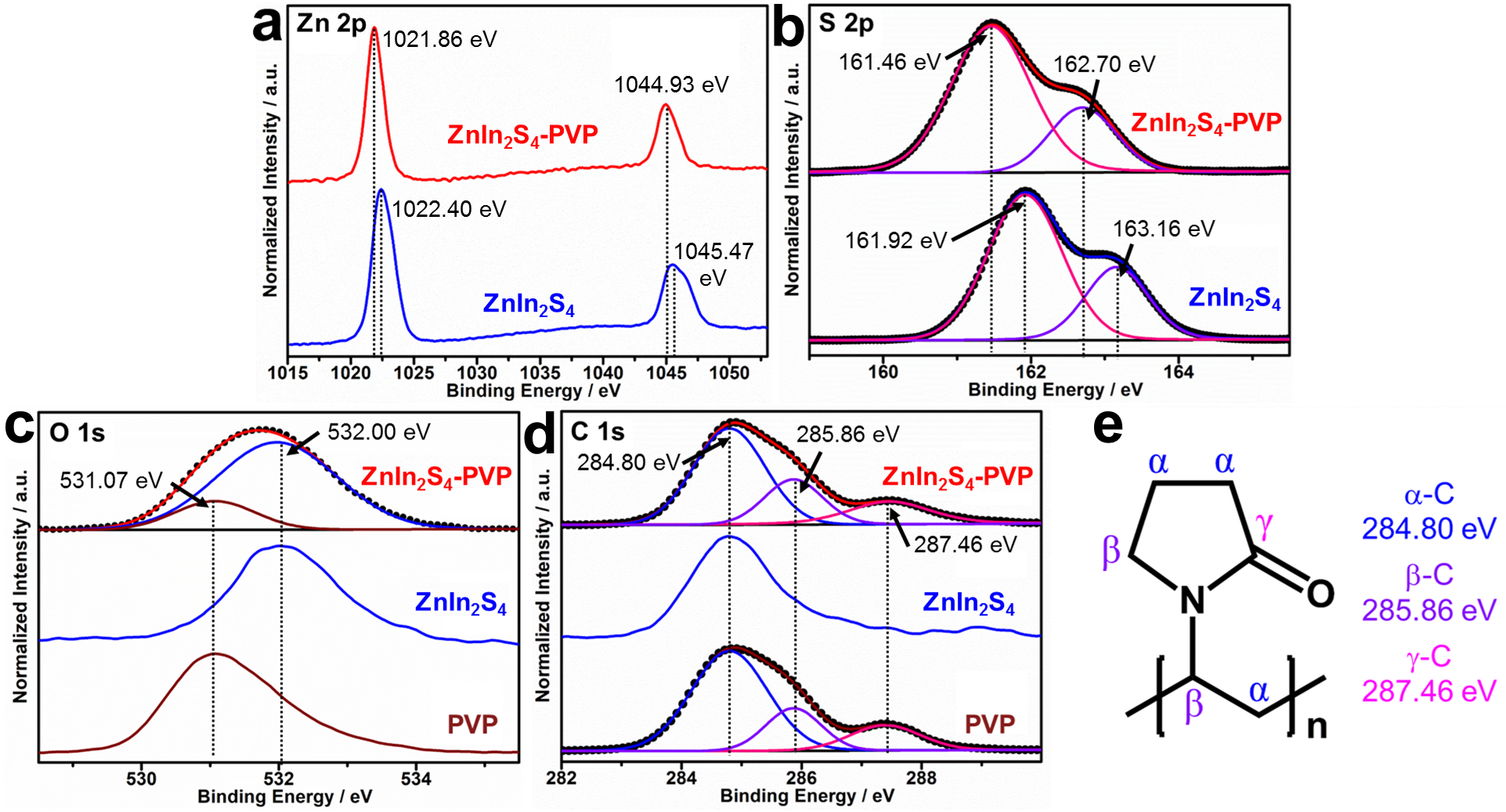


**Supplementary Figure 6.** XPS analysis :(a) Zn 2p spectra; (b) S 2p spectra; (c) O 1s spectra; (d) C 1s spectra. (e) Structural formula of PVP and the corresponding binding energy of α-C, β-C, γ-C. As for Zn 2p and S 2p spectra, ZnIn_2_S_4_-PVP exhibits approximately 0.54 and 0.46 eV blue-shift relative to the ZnIn_2_S_4_, respectively. In O 1s spectra, the peaks at 531.07 and 532 eV represent carbonyl oxygen in PVP and surface adsorbed oxygen, respectively [2]. As for C 1s spectra, the peaks at 284.8, 285.86, and 287.46 eV assign to α-C (including the standard reference carbon), β-C, and γ-C of PVP, respectively.


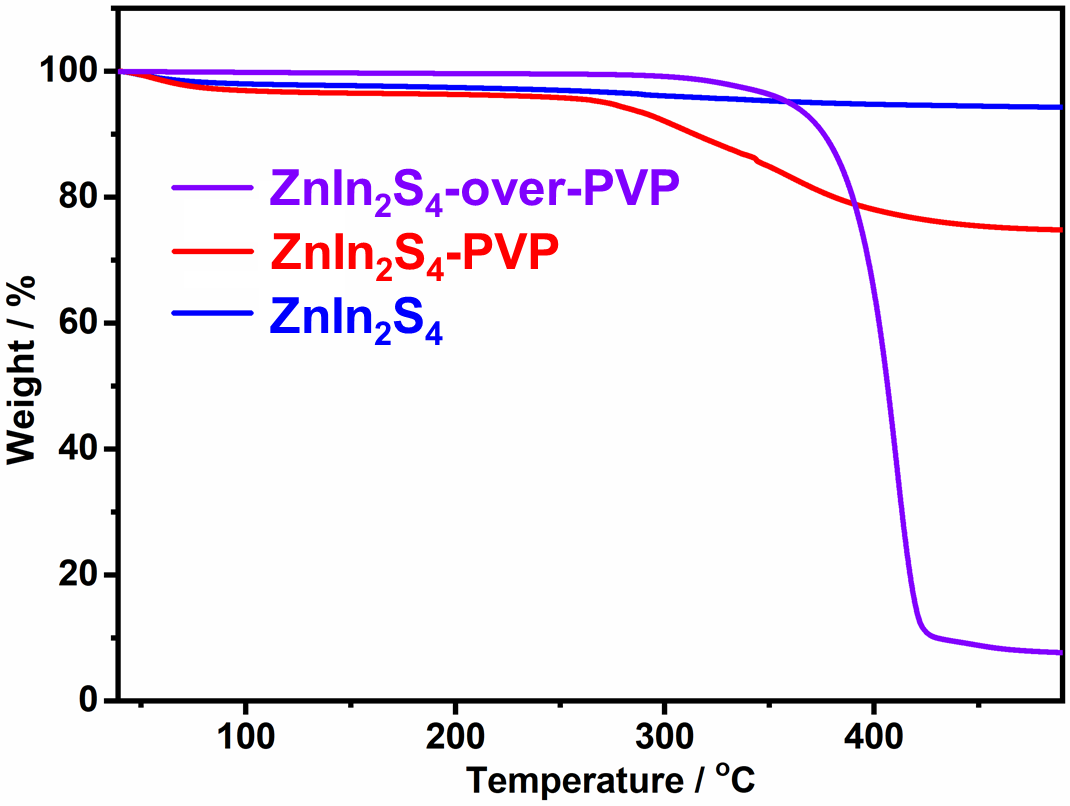


**Supplementary Figure 7.** TGA of ZnIn_2_S_4_, ZnIn_2_S_4_-PVP and ZnIn_2_S_4_-over-PVP. Thermogravimetric analysis (TGA) of ZnIn_2_S_4_-PVP and ZnIn_2_S_4_-over-PVP nanosheets displayed an obvious decrease from ~270 to ~430 °C, which could be attributed to decomposition of the introduced PVP [3]. Additionally, ZnIn_2_S_4_-over-PVP nanosheets contain about 90 % of PVP in weight.


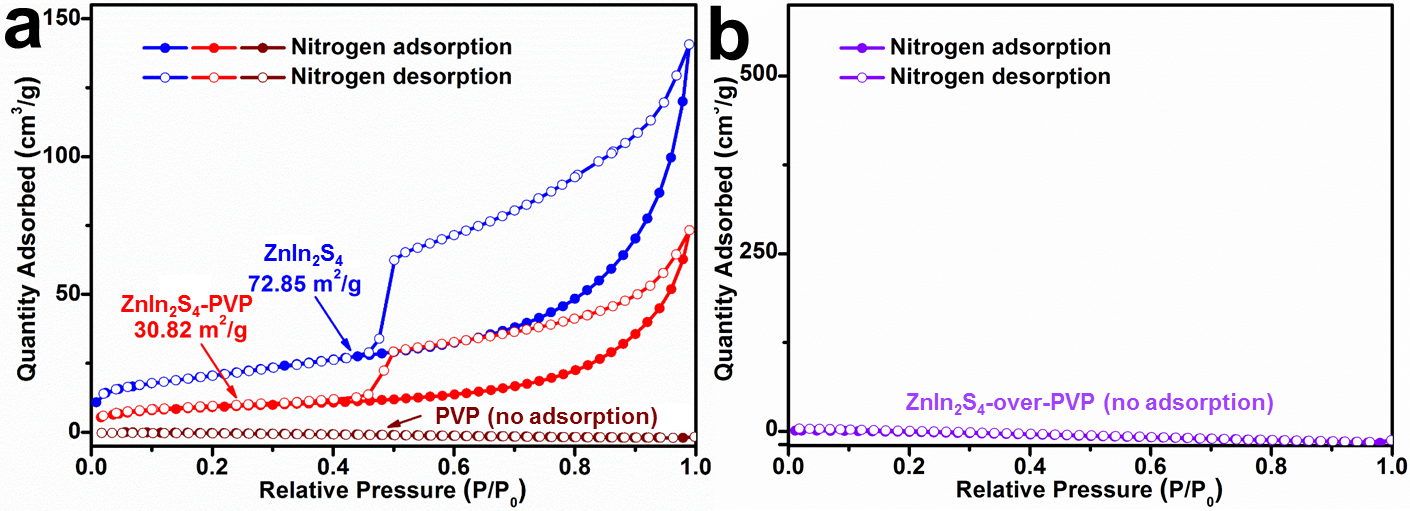


**Supplementary Figure 8.** Nitrogen absorption-desorption isotherms at 77 K. ZnIn_2_S_4_-over-PVP nanosheets exhibited no adsorption, proving that over covered PVP entirely stopped this adsorption process.


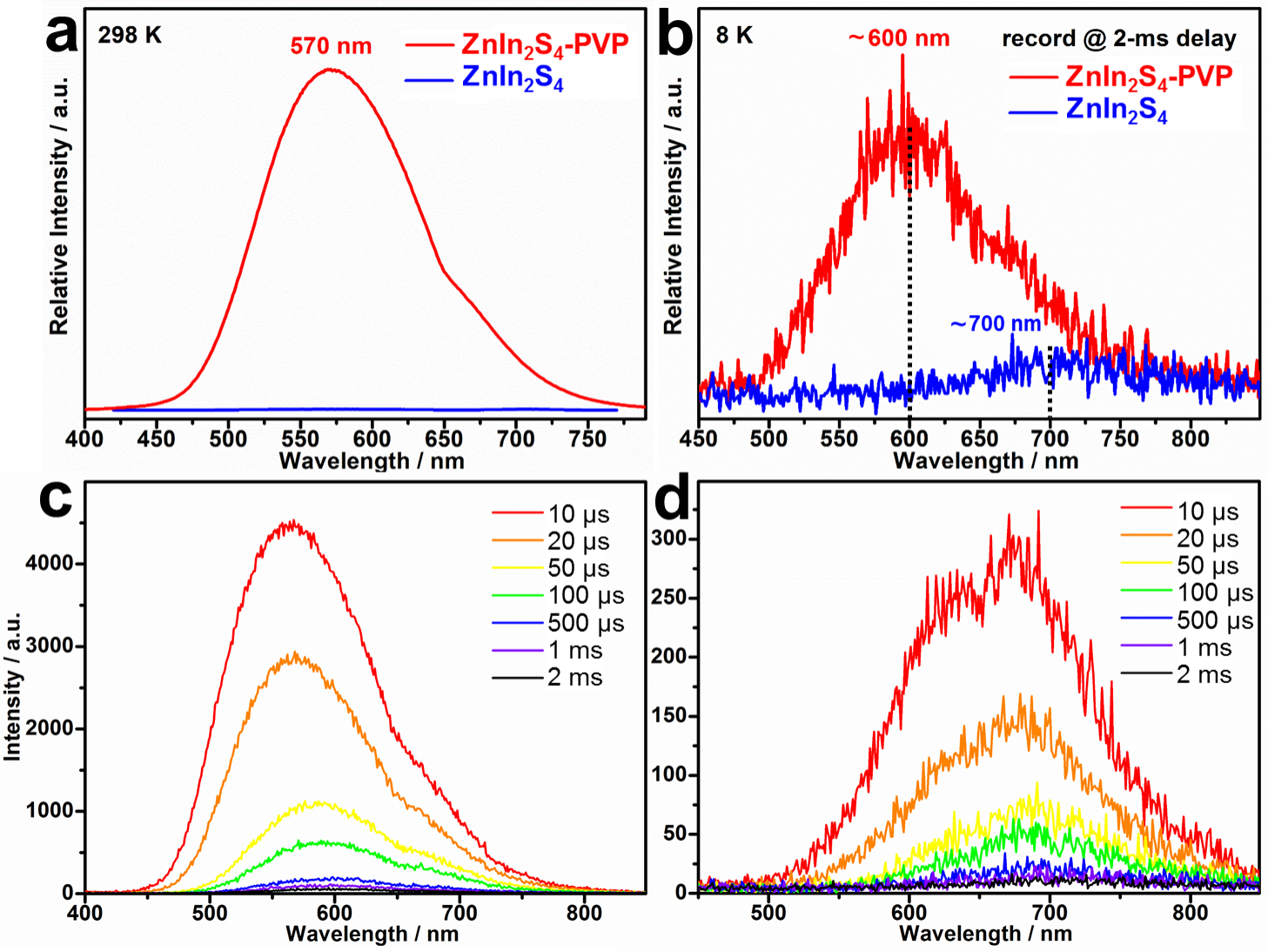


**Supplementary Figure 9.** (a) Room-temperature prompt fluorescence (PF); (b) low-temperature (8 K) steady-state phosphorescence recorded at a delayed time of 2 ms; low-temperature (8 K) steady-state phosphorescence recorded at different delayed times of (c) ZnIn_2_S_4_-PVP and (d) pristine ZnIn_2_S_4_ (all excited at 370 nm).


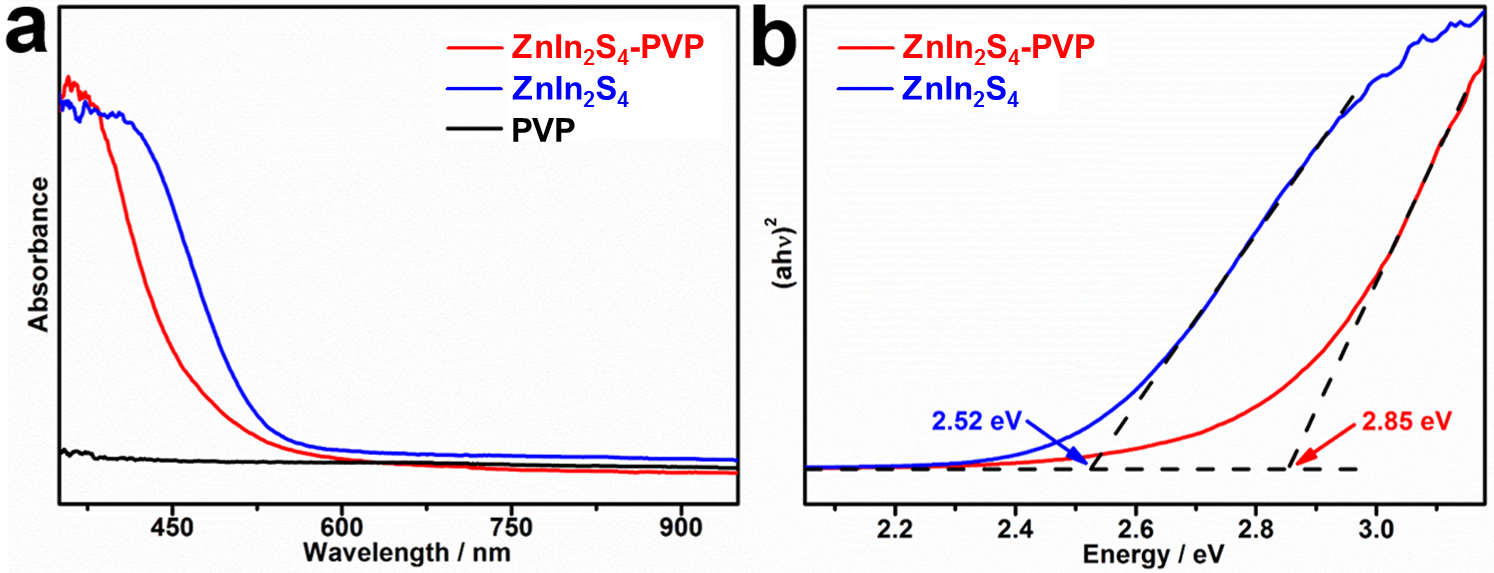


**Supplementary Figure 10.** (a) UV−Vis absorption spectra and (b) the corresponding Tauc plots. Both ZnIn_2_S_4_-PVP and ZnIn_2_S_4_ can efficiently absorb visible light.


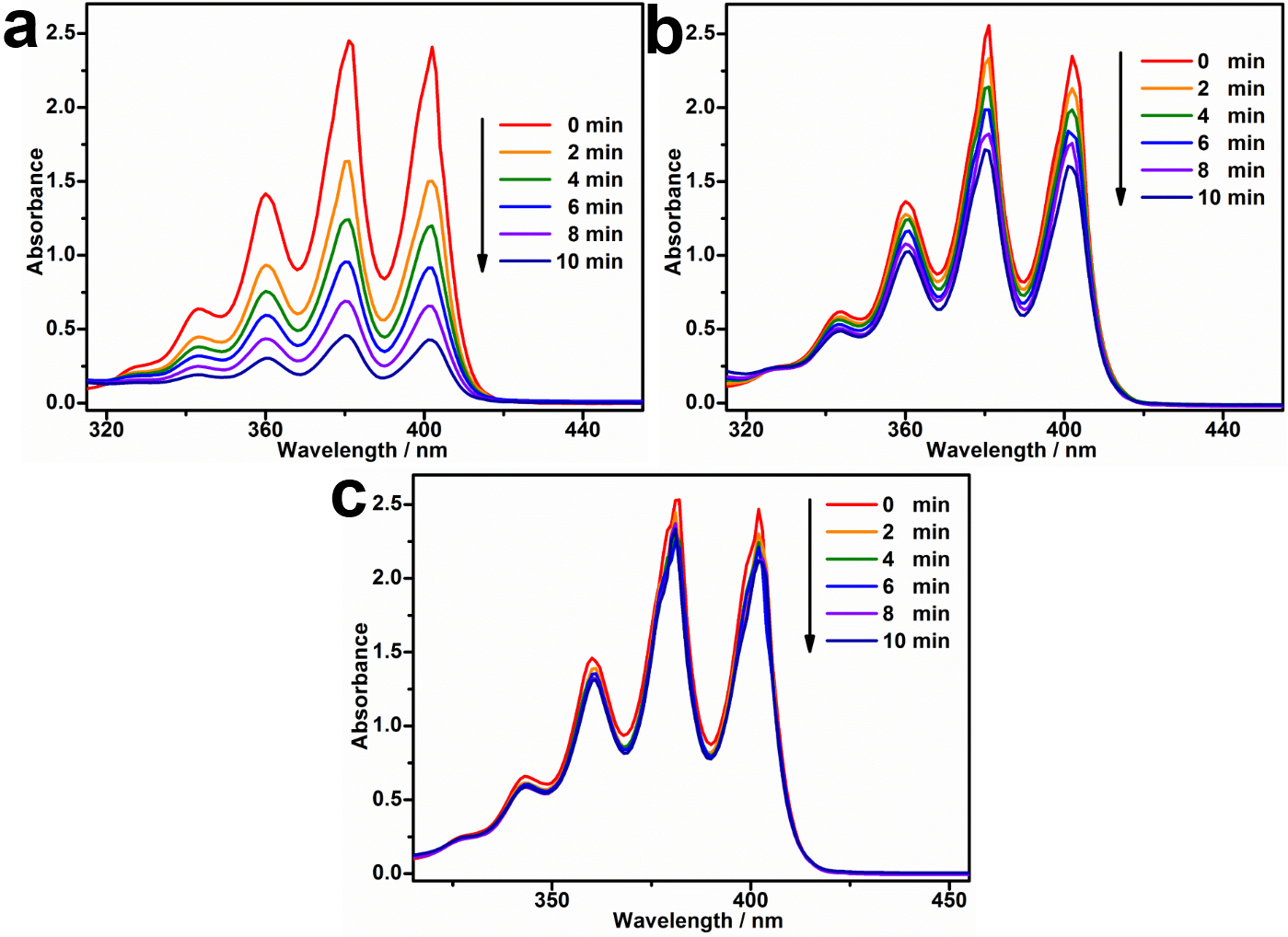


**Supplementary Figure 11.** The oxidation of ABDA evaluated by UV−Vis measurements (the absorbance from ~320 nm to ~450 nm) at different time intervals: (a) ZnIn_2_S_4_-PVP, O_2_, 1 atm; (b) ZnIn_2_S_4_, O_2_, 1 atm; (c) ZnIn_2_S_4_-PVP, N_2_, 1 atm. The degradation of ABDA indicated the ^1^O_2_ generation ability of ZnIn_2_S_4_-PVP and ZnIn_2_S_4_. For ZnIn_2_S_4_-PVP at atmosphere of N_2_, the absorbance showed no obvious decrease, confirming the vital role of O_2_ for ^1^O_2_ generation.


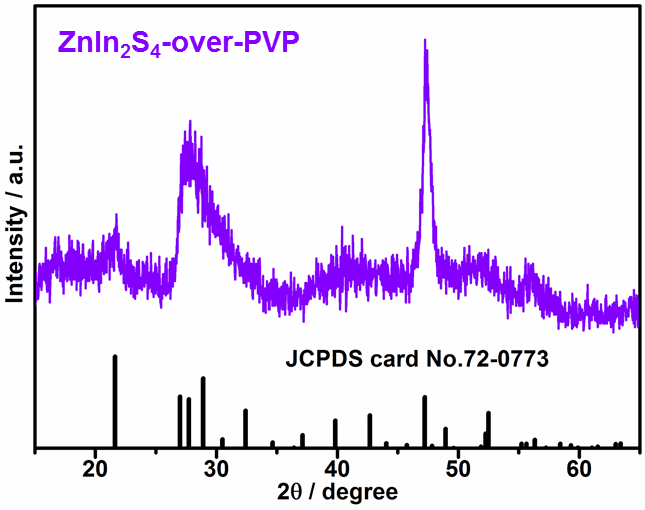


**Supplementary Figure 12.** XRD patterns of ZnIn_2_S_4_-over-PVP.


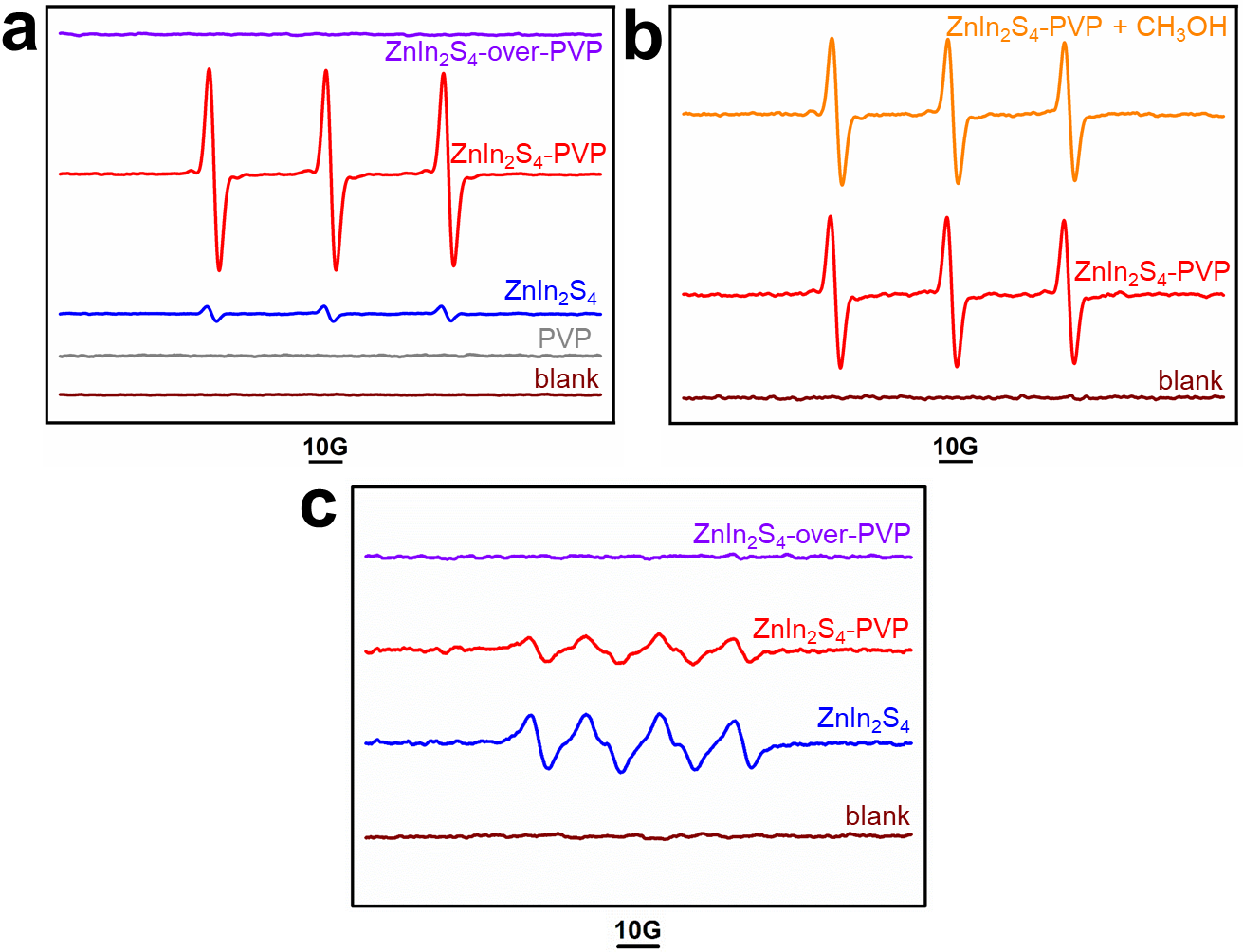


**Supplementary Figure 13.** ESR spectra in the presence of (a and b) TEMP and (c) DMPO. As seen from the triplet signal which identified photogenerated ^1^O_2_ in Fig. S13a and b, no obvious triplet signal was detected by ZnIn_2_S_4_-over-PVP and PVP; the addition of methanol (effective hole scavenger) did not suppress ^1^O_2_ generation. After changing the TEMP into 5,5-dimethyl-1-pyrroline-N-oxide (DMPO) in ESR trapping measurements, generated O_2_^•−^ will display a signal associated with DMPO–OOH which is a spin derivative of DMPO–O_2_^•−^. In Fig. S13c, ZnIn_2_S_4_ showed a higher signal than ZnIn_2_S_4_-PVP; no obvious signal was detected by ZnIn_2_S_4_-over-PVP.


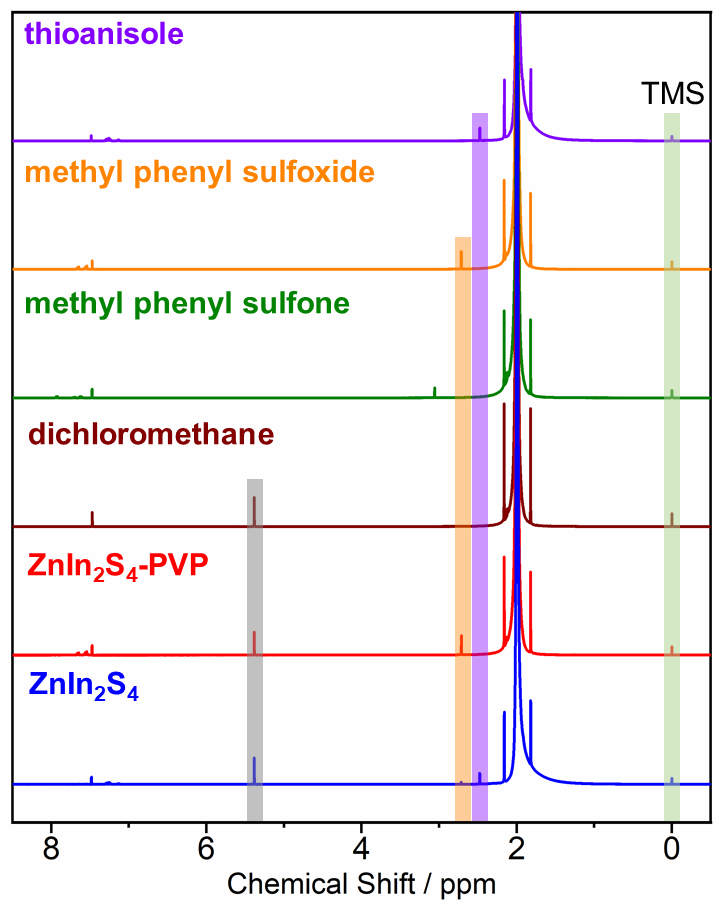


**Supplementary Figure 14.** Representative ^1^H NMR spectra of thioanisole, methyl phenyl sulphoxide, methyl phenyl sulphone, dichloromethane in acetonitrile (CH_3_CN) solution, and products of ZnIn_2_S_4_-PVP after photocatalytic reaction (Table 1, entry 1), products of ZnIn_2_S_4_ after photocatalytic reaction (Table 1, entry 3).


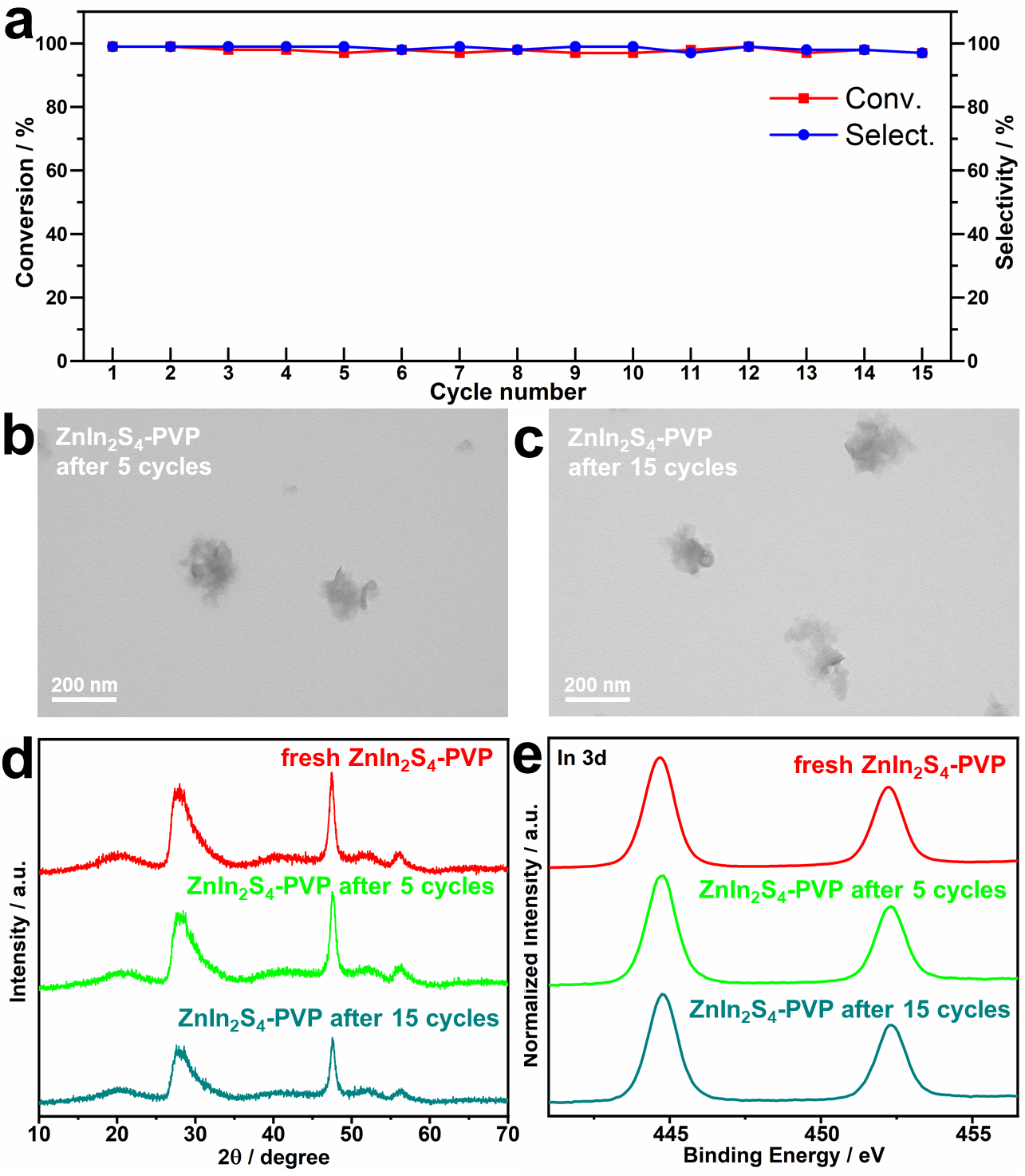


**Supplementary Figure 15.** (a) Cycling tests of photocatalytic sulphoxidation of sulphides for ZnIn_2_S_4_-PVP. TEM images of ZnIn_2_S_4_-PVP after (b) 5 and (c) 15 cycles of reaction. (d) XRD patterns and (e) In 3d spectra for ZnIn_2_S_4_-PVP after 5 and 15 cycles of reaction. All tests suggest that ZnIn_2_S_4_-PVP possess excellent stability even after 15 catalytic cycles.


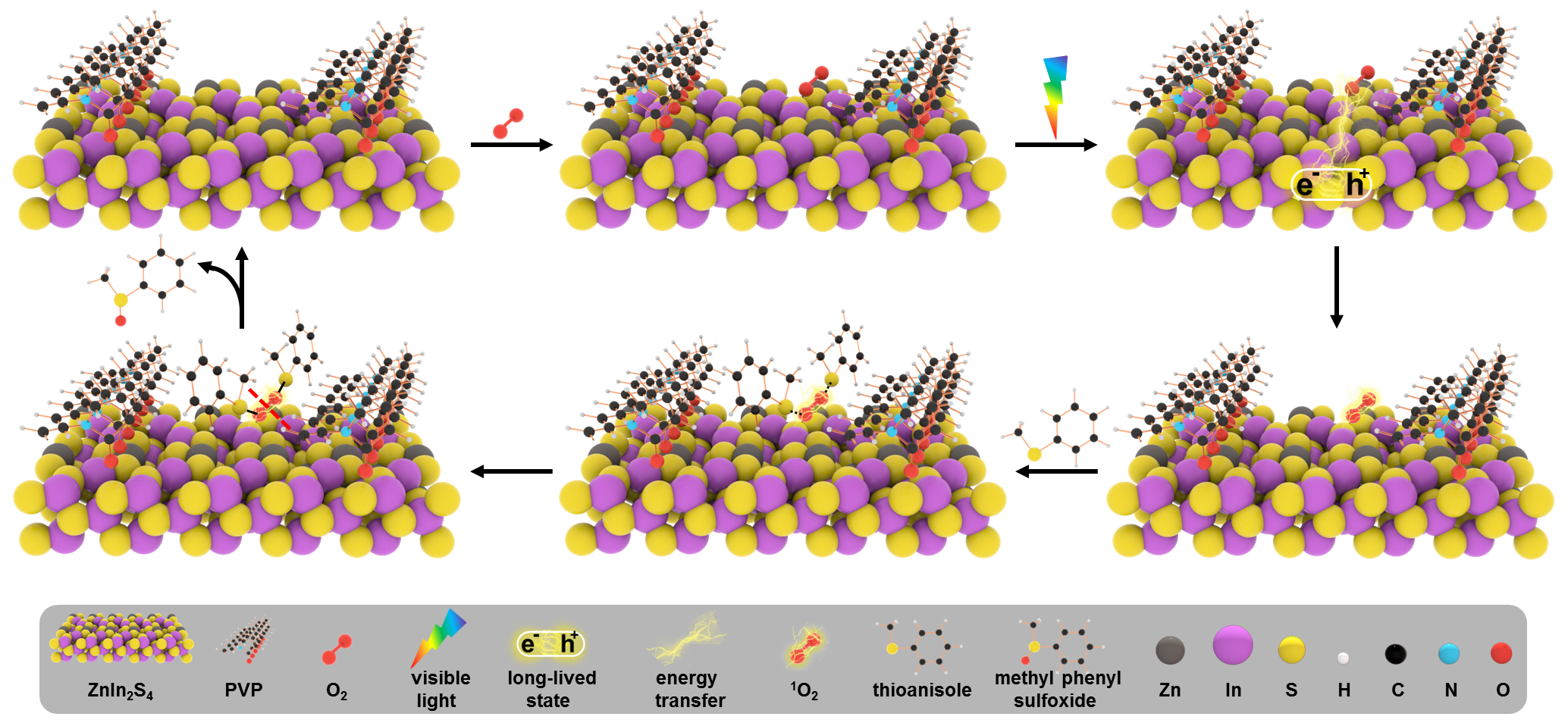


**Supplementary Figure 16.** Proposed mechanism of photocatalytic sulphoxidation of sulphides. If the R_2_ group is larger than methyl (like cyclopropyl and phenyl), the conversion rates of catalytic process will be reduced during steric hindrance.

**Supplementary Table 1.** Sulphoxidation of sulphides.^a)^

| Entry | R_1_ | R_2_ | Time / h | ZnIn_2_S_4_-PVP | | ZnIn_2_S_4_ | |
| --- | --- | --- | --- | --- | --- | --- | --- |
|  |  |  |  | Con.^b)^ | Sel.^c)^ | Con. | Sel. |
| 1 | Ph | cyclopropyl | 16 | 44 | 99 | 15 | 99 |
| 2 | Ph | Ph | 16 | 17 | 91 | 4.0 | 88 |

a) Reaction conditions: catalyst (20 mg), substrate (0.1 mmol), acetonitrile (4 mL), xenon lamp (300 W) equipped with a 400 nm cutoff filter, 298 K, O_2_ (1 atm). b) Determined by NMR analyses using dichloromethane as the internal standard, mol %. c) Selectivity = yield/conversion, mol %.

**References**

[1] Safo IA, Werheid M and Dosche C *et al.* The role of polyvinylpyrrolidone (PVP) as a capping and structure-directing agent in the formation of Pt nanocubes. *Nanoscale Adv* 2019; **1**: 3095-106.

[2] Tu Y, Chen S and Li X *et al.* Control of oxygen vacancies in ZnO nanorods by annealing and their influence on ZnO/PEDOT: PSS diode behaviour. *J Mater Chem C* 2018; **6**: 1815-21.

[3] Nuansing W, Ninmuang S and Jarernboon W *et al.* Structural characterization and morphology of electrospun TiO_2_ nanofibers. *Mater Sci Eng, B* 2006; **131**: 147-55.
